# Synthesis and Storage Stability of Diisopropylfluorophosphate

**DOI:** 10.1155/2016/3190891

**Published:** 2016

**Authors:** Derik R. Heiss, Donald W. Zehnder, David A. Jett, Gennady E. Platoff, David T. Yeung, Bobby N. Brewer

**Affiliations:** 1 Battelle Memorial Institute, 505 King Avenue, Columbus, OH 43201, USA; 2 National Institute of Neurological Disorders and Stroke, National Institutes of Health, 6001 Executive Boulevard, Rockville, MD 20852, USA; 3 National Institute of Allergy and Infectious Diseases, National Institutes of Health, 5601 Fishers Lane, Rockville, MD 20892, USA

## Abstract

Diisopropylfluorophosphate (DFP) is a potent acetylcholinesterase inhibitor commonly used in toxicological studies as an organophosphorus nerve agent surrogate. However, LD_50_ values for DFP in the same species can differ widely even within the same laboratory, possibly due to the use of degraded DFP. The objectives here were to identify an efficient synthesis route for high purity DFP and assess the storage stability of both the in-house synthesized and commercial source of DFP at the manufacturer-recommended storage temperature of 4°C, as well as −10°C and −80°C. After 393 days, the commercial DFP stored at 4°C experienced significant degradation, while only minor degradation was observed at −10°C and none was observed at −80°C. DFP prepared using the newly identified synthesis route was significantly more stable, exhibiting only minor degradation at 4°C and none at −10°C or −80°C. The major degradation product was the monoacid derivative diisopropylphosphate, formed via hydrolysis of DFP. It was also found that storing DFP in glass containers may accelerate the degradation process by generating water *in situ* as hydrolytically generated hydrofluoric acid attacks the silica in the glass. Based on the results here, it is recommended that DFP be stored at or below −10°C, preferably in air-tight, nonglass containers.

## 1. Introduction

**Figure 1:**
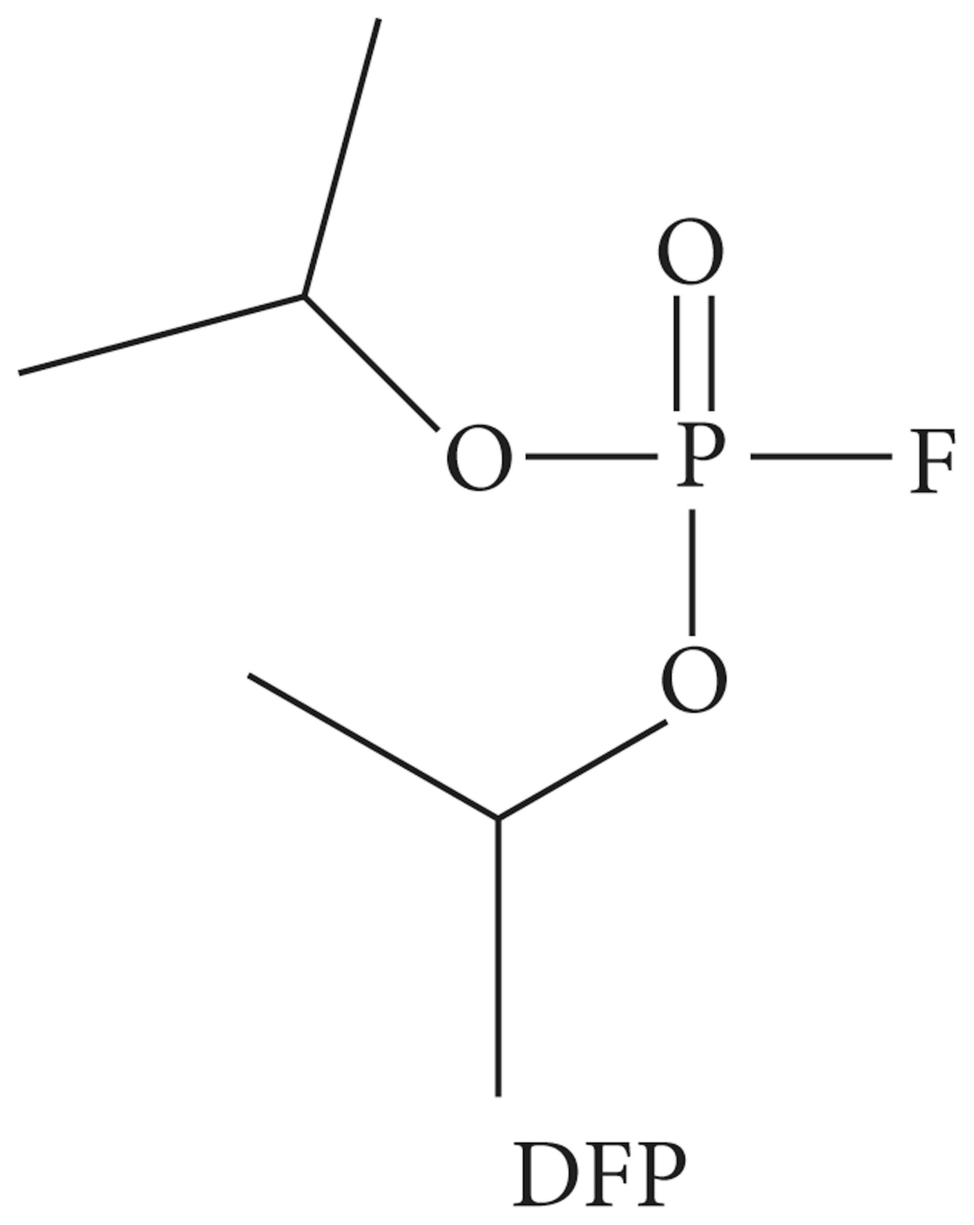
Diisopropylfluorophosphate (DFP).

Diisopropylfluorophosphate (DFP, see Figure 1) is a powerful neurotoxin often used in research studies as a surrogate for organophosphorus nerve agents such as sarin (GB) and soman (GD) due to its ability to effectively inhibit the enzyme acetylcholinesterase [1–3]. However, significant variability has been observed in toxicological studies using commercially available DFP. For example, published subcutaneous LD_50_ values range from 0.0027 mg/kg to 6.4 mg/kg in the mouse model [4–6].

The storage stability of DFP, information especially useful in support of long-term evaluations, has not been documented. However, anecdotal information suggests that DFP degrades upon storage, and it is speculated that the wide range in reported LD_50_ values may be caused by the use of impure DFP. As such, proper storage of DFP for use in analytical study is imperative.

The objectives of this study were to synthesize high purity DFP and then evaluate the storage stability of both the synthesized DFP and a commercial source of DFP [7]. The manufacturer-recommended storage temperature for commercially available DFP is 4°C [8]. Therefore, this study compared DFP stored at 4°C against material stored at other common laboratory storage temperatures, −10°C and −80°C.

## 2. Materials and Methods

### 2.1. Chemicals

Analytical grade DFP was procured from Sigma-Aldrich. Diisopropylphosphate (DIPP) was obtained from PolyOrg, Inc. All solvents and synthesis reagents were purchased from Sigma-Aldrich and were of ACS grade or better.

### 2.2. Instrumentation

Nuclear magnetic resonance (NMR) data were collected using a Bruker Advance 500 FT-NMR with an operating field of 11.75 Tesla. Fourier-transform infrared (FT-IR) spectra were collected with a Digilab FTS-7000 with UMA-600 microscope using NaCl plating technique. Gas chromatography-mass spectrometry (GC-MS) data were obtained using an Agilent 6890 gas chromatograph with Model 5973N mass spectrometer. X-ray diffraction (XRD) data were recorded using a Rigaku Ultima IV diffractometer.

### 2.3. Synthesis of DFP

Potassium fluoride (8.17 g, 140.9 mmol) was added to 1,3-dichloro-5,5-dimethylhydantoin in acetonitrile (300 mL) and then stirred at room temperature for one hour. Diisopropyl phosphite (18 g, 108 mmol) in acetonitrile (100 mL) was added to the mixture all at once and then stirred for 30 minutes. The resulting white precipitate was removed by filtration over diatomaceous earth followed by a 0.45 *μ*m PTFE membrane filter. The concentrated crude product was purified by distillation (bp. 63°C, 8 mmHg) affording 13.7 g (68% yield) of a clear, colorless liquid with a purity of 99%, as determined by ^1^H and ^31^P NMR.

### 2.4. DFP Stability Study

Two sources of DFP, one procured from Sigma-Aldrich and one synthesized as described above, were each divided into approximately 30 mg, single-use aliquots in clear glass vials with PTFE-lined screw caps. The vials were stored in the dark at either 4°C, −10°C, or −80°C (±2°C) surrounded with cold packs inside coolers to prevent unwanted thermal cycling. At approximately 2-week intervals, duplicate sacrificial vials of each material at each storage temperature were removed and warmed to room temperature in a desiccator. The samples were then dissolved in acetonitrile-*d*_3_ or other appropriate solvents and analyzed by ^31^P NMR to determine purity.

Degradation products were determined using ^31^P NMR by dissolving an aliquot of DFP in acetonitrile-*d*_3_. In addition, a second sample of DFP was analyzed in neat form by FT-IR while a third portion of DFP was extracted with methylene chloride, centrifuged to remove undissolved solids, and analyzed by GC-MS. The solid remaining from this aliquot was rinsed with methylene chloride, dried, and analyzed by FT-IR and XRD.

## 3. Results and Discussion

### 3.1. Synthesis of DFP

Dialkylfluorophosphates, including DFP, are traditionally synthesized from the corresponding chlorophosphate using a fluorinating agent [9–11]. In many cases, the reaction is slow and often does not go to completion, leaving unreacted starting material remaining as an impurity.

**Figure 2:**
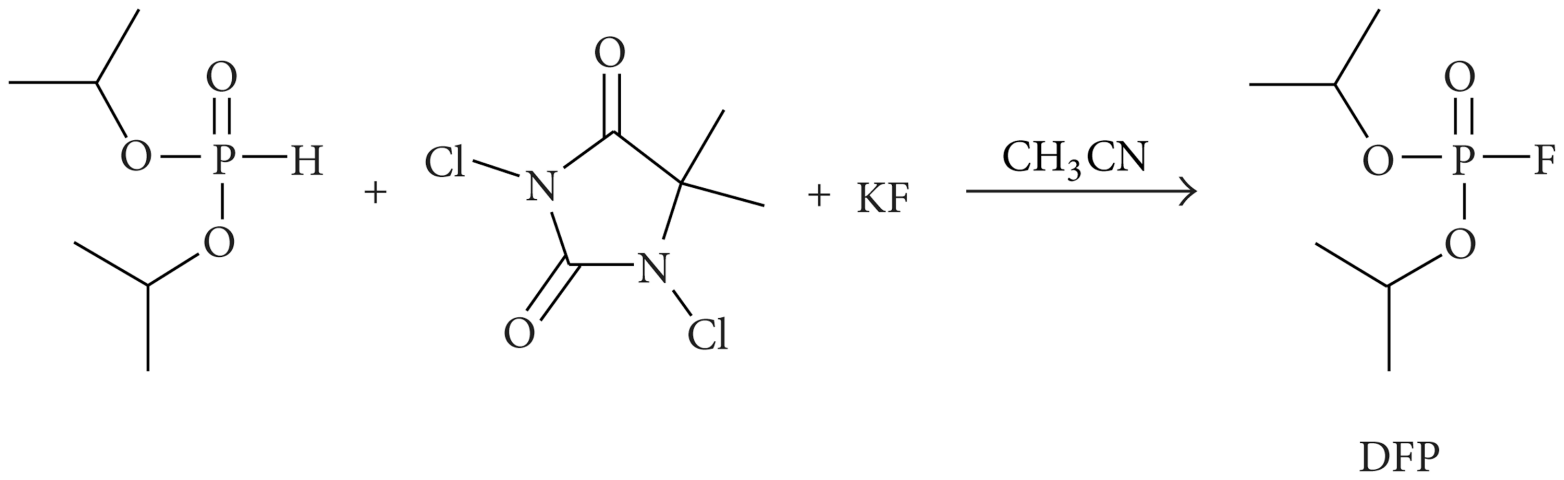
One-pot synthesis of DFP.

For this study, a previously reported one-pot synthesis method [12] was modified to produce high purity DFP from diisopropyl phosphite using a mixture of KF and 1,3-dichloro-5,5-dimethylhydantoin (see Figure 2). The intermediate diisopropyl chlorophosphate formed *in situ* is rapidly converted to the corresponding fluorophosphate. Vacuum distillation of the resultant reaction mixture produced DFP in 68% yield with a purity of 99%, as determined by ^1^H and ^31^P NMR.

### 3.2. Storage Stability of DFP

**Figure 3:**
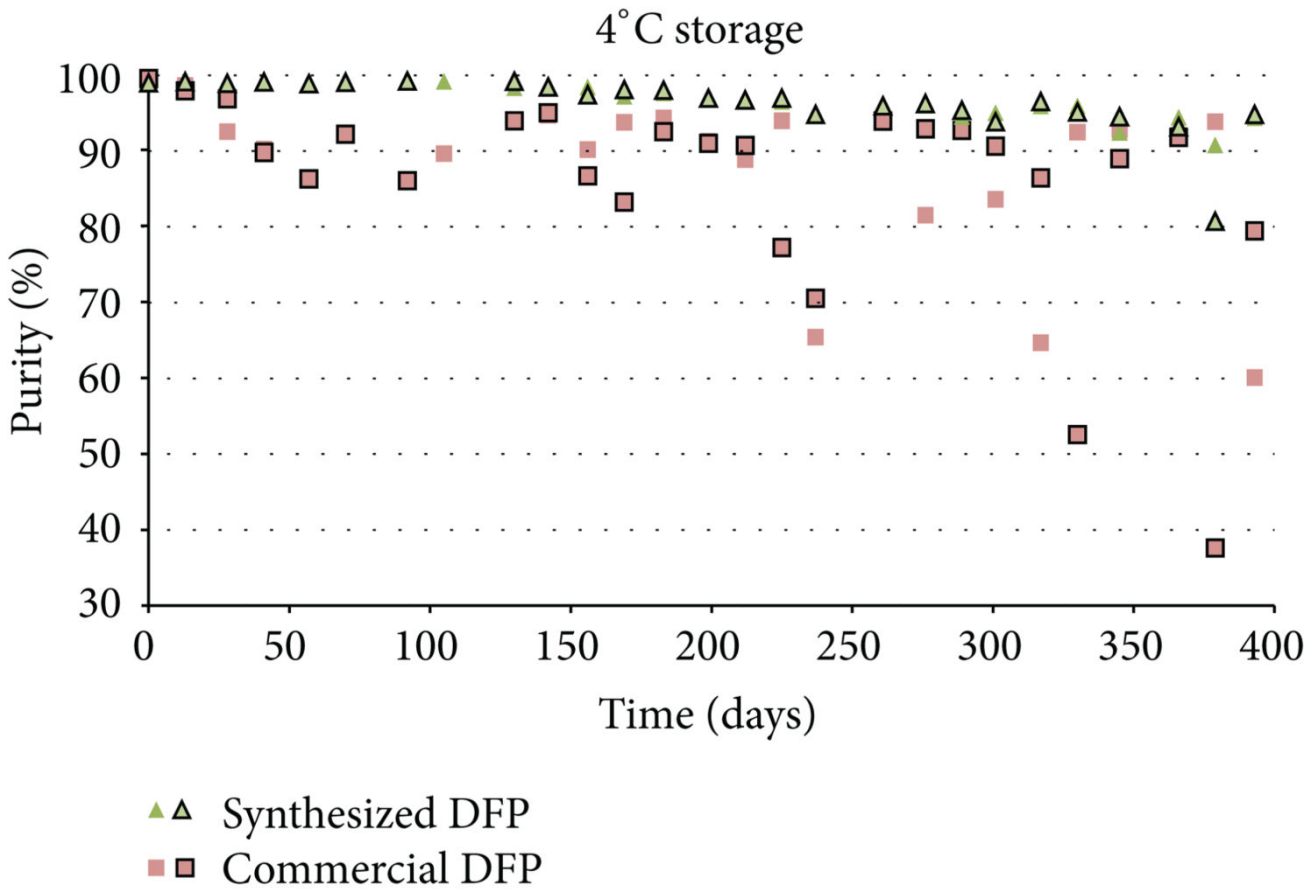
Purity results for synthesized DFP and commercial DFP when stored at 4°C.

**Figure 4:**
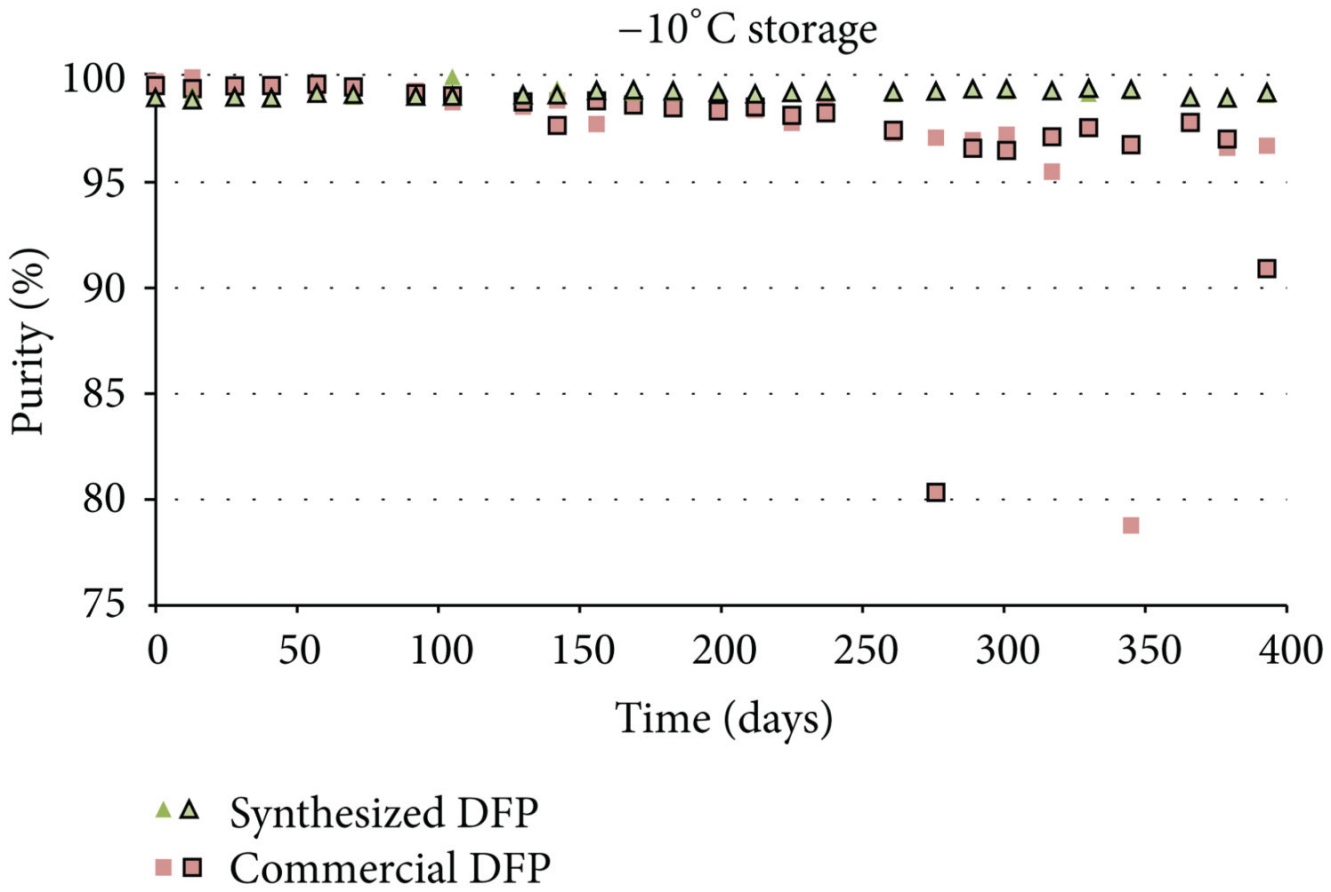
Purity results for synthesized DFP and commercial DFP when stored at −10°C.

**Figure 5:**
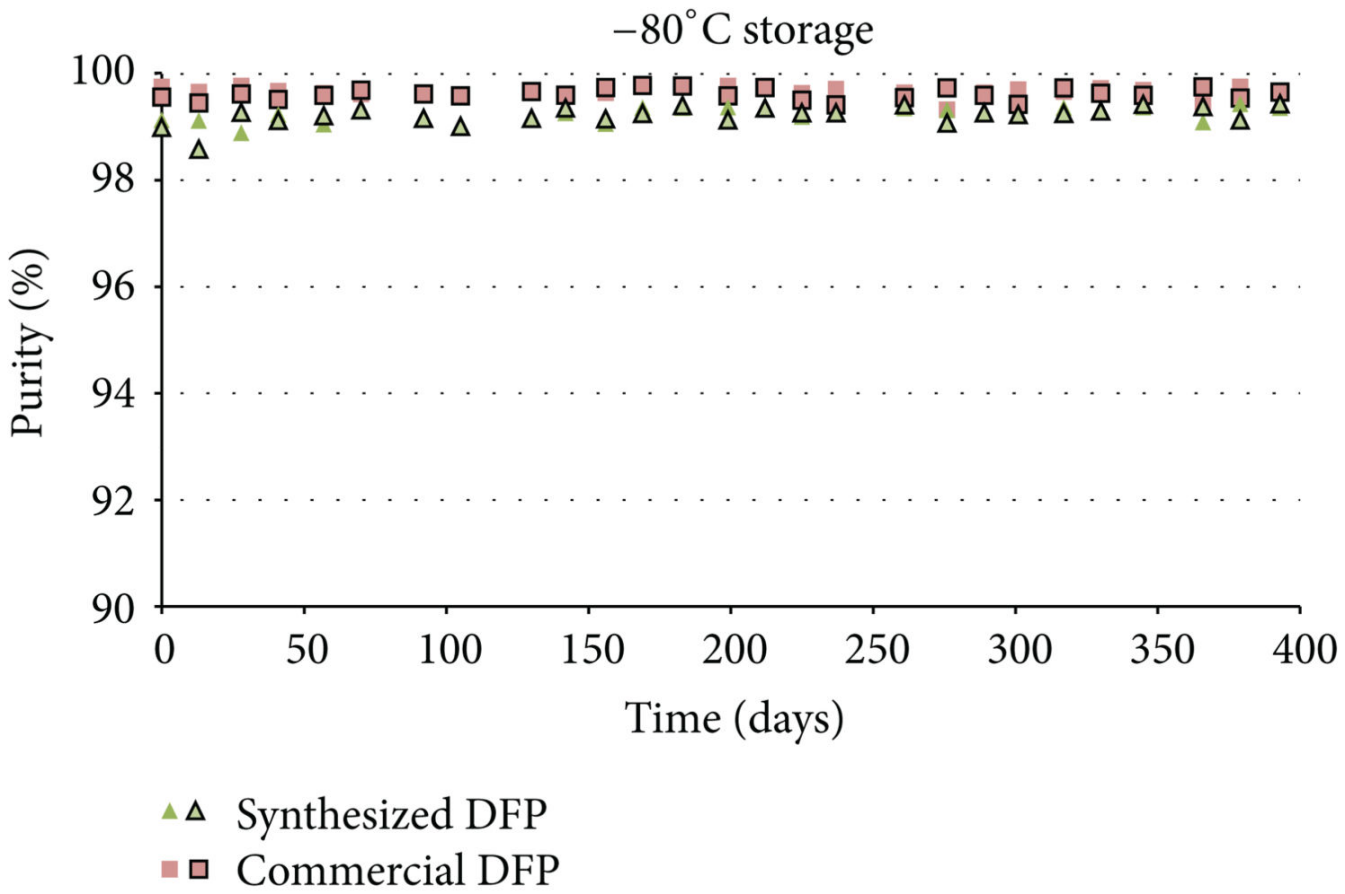
Purity results for synthesized DFP and commercial DFP when stored at −80°C.

Both the synthesized DFP and the commercially acquired DFP were analyzed at the beginning of the study (day 0) and found to have initial purity values of >99% by ^31^P NMR. Purity assessments were then conducted in duplicate at approximately 2-week intervals over the course of 13 months (393 days) to evaluate the stability of DFP under each of the three storage conditions. Purity results for each replicate analysis are presented in Figures 3, 4, and 5. DFP purity data are presented as outlined and nonoutlined green triangles or red squares representing individual synthesized and commercial samples, respectively.

As the purity results indicate, the commercial source of DFP degraded significantly when stored at 4°C, while degradation was markedly slower when stored at −10°C. In this study, 88% of the vials of commercial DFP stored at 4°C had degraded below 95% purity within 393 days, while only 6% of the vials stored at −10°C fell below 95% purity. No degradation was observed in vials stored at −80°C. By contrast, the synthesized DFP was considerably more stable. Only 21% of the vials of synthesized DFP stored at 4°C and none of the vials stored at −10°C or −80°C had degraded to less than 95% purity within 393 days. The cause of the disparity in degradation rates observed between the two sources of DFP was not investigated for this study but can likely be attributed to differences in the impurity profiles introduced during synthesis.

**Figure 6:**
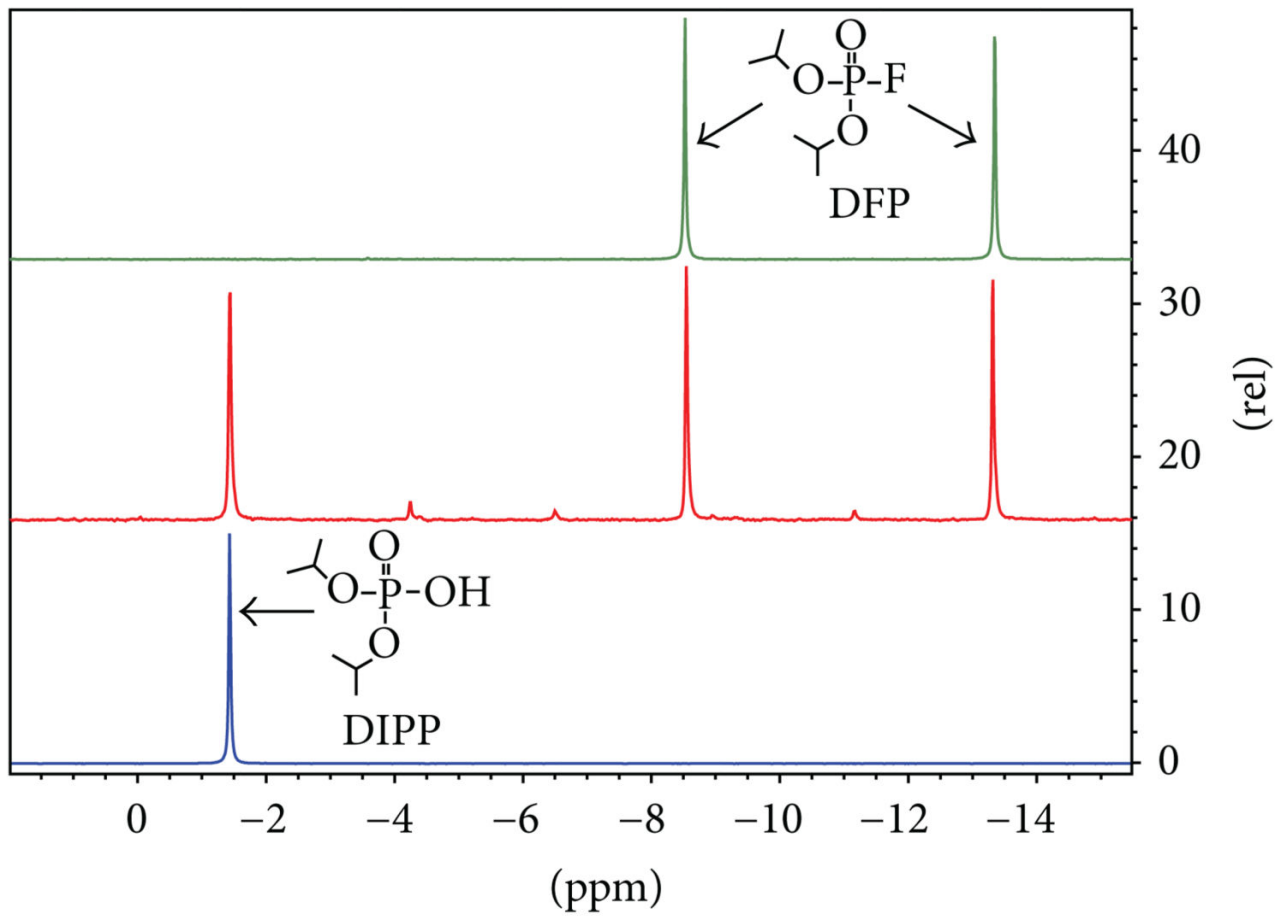
^31^P NMR spectra of pure DFP (top), partially degraded commercial DFP (middle), and a pure DIPP standard (bottom).

**Figure 7:**
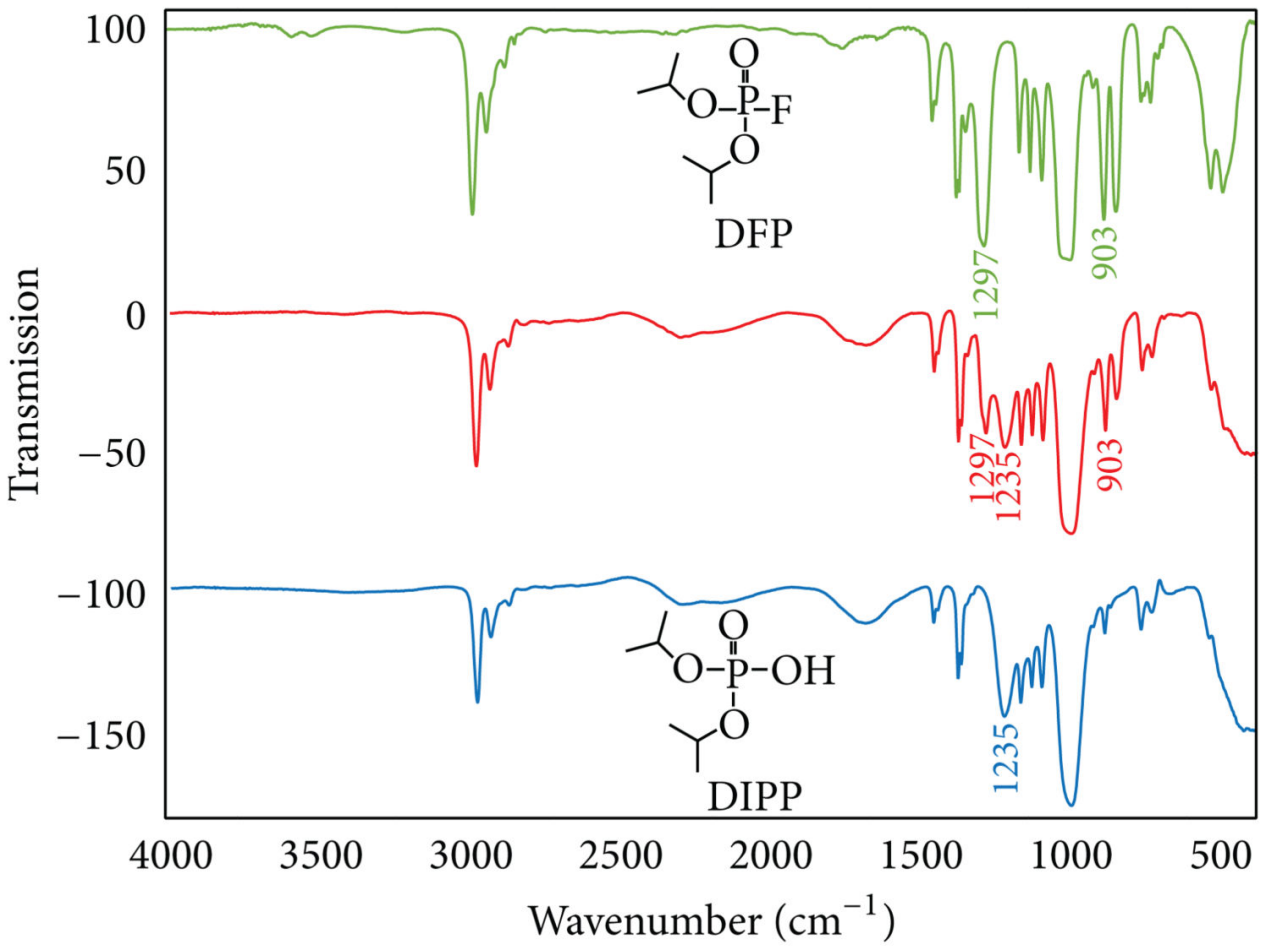
FT-IR spectra of pure DFP (top), partially degraded commercial DFP (middle), and a pure DIPP standard (bottom).

As expected, the major degradation product of DFP was found to be the hydrolysis product diisopropylphosphate (DIPP). This was confirmed upon comparison of the degraded material to a known standard of DIPP using ^31^P NMR and FT-IR spectroscopy (see Figures 6 and 7). In addition, a small amount of triisopropylphosphate had formed, as indicated by GC-MS analysis.

**Figure 8:**
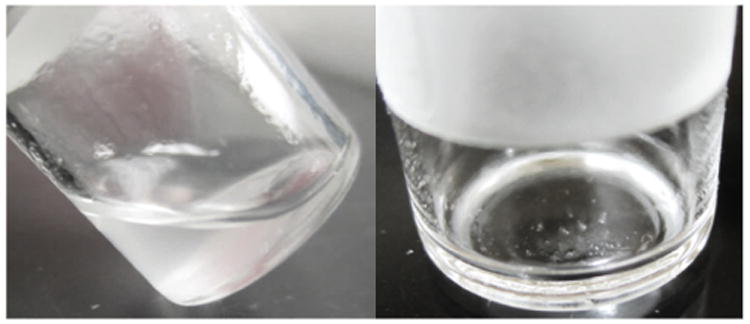
White solid observed in partially degraded DFP.

A white solid began to form in the neat DFP as purity fell to approximately 90% or below (see Figure 8). The presence of the solid was somewhat confounding, as both DFP and its hydrolysis products are liquids at room temperature.

The solid was identified using FT-IR and XRD as a hexafluorosilicate salt. Its presence in the degraded DFP can likely be explained by a secondary reaction between hydrofluoric acid (HF), formed as DFP hydrolyzes, and silica from the glass storage vials. HF is known to react with silicate glass and is commonly used as a wet chemical etchant in industrial processes [13].

**Figure 9:**
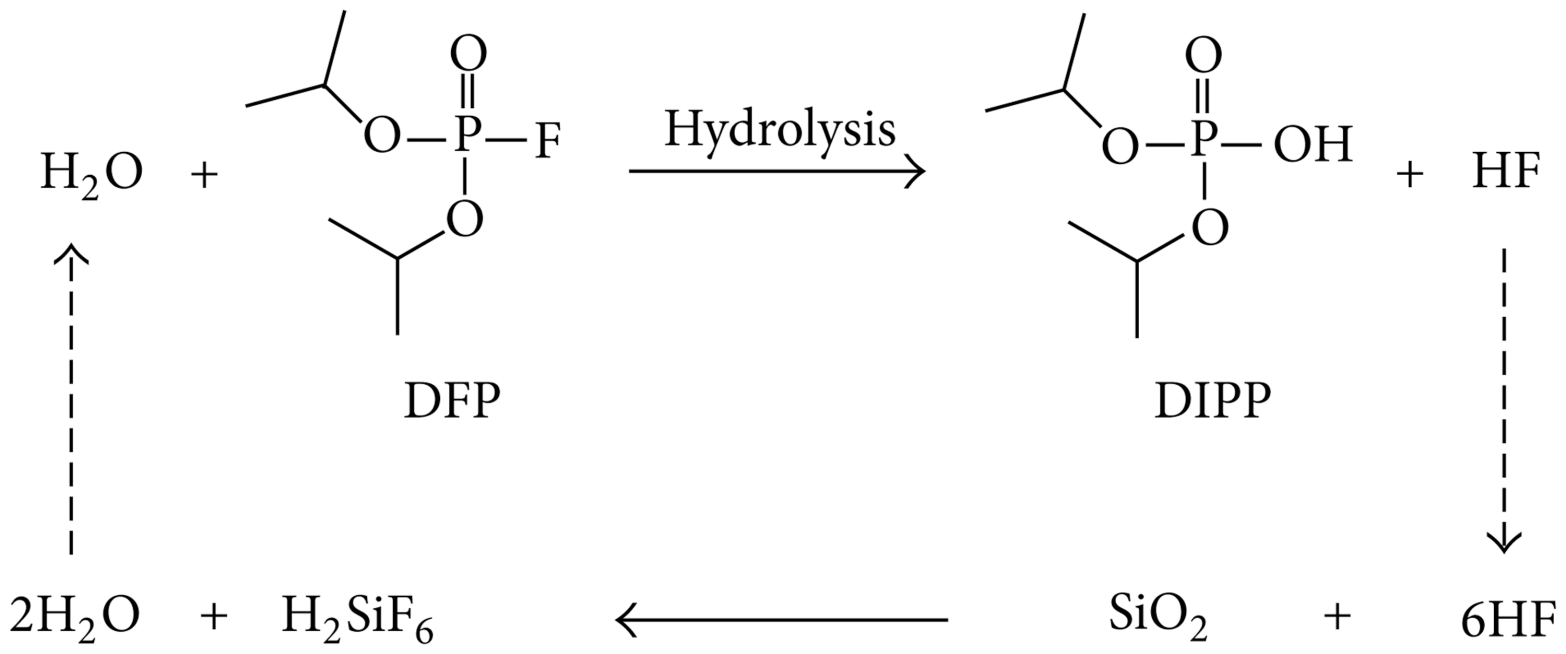
Autocatalytic hydrolysis of DFP when stored in glass vials.

Interestingly, this side reaction may be responsible for accelerating the degradation of DFP in two ways: (1) by consuming HF, thus driving the equilibrium toward the products side of the hydrolysis reaction (i.e., to the right), and (2) by generating water *in situ*, resulting in a self-sustaining hydrolysis cycle (Figure 9).

In the initial stages of DFP degradation, hydrolysis is the primary reaction mechanism and appears to proceed according to standard first- or second-order kinetics. However, after a certain induction period elapses and the material degrades further, the HF produced may begin to react with silica in the glass forming the insoluble hexafluorosilicate salt (white solid) and water. The water generated by the reaction can then initiate further hydrolysis, resulting in an autocatalytic reaction cycle that ultimately accelerates the degradation of DFP.

Because DFP degrades rapidly once hydrolysis becomes self-sustaining, small differences in surface reactivity of the glass vials or the amount of surface area exposed to the degraded DFP can likely lead to large discrepancies in purity values among replicates of the same material. This might explain the variability observed in some of the purity results for the same material, most notably the commercial DFP stored at 4°C (see Figure 3). For this study, it was assumed that all vials of the same material stored under the same conditions would behave similarly. If this assumption does not hold, which appears to be the case here, variability can be introduced even if all other parameters are held constant. As such, it is recommended that DFP be stored in nonglass containers.

Similar storage issues resulting from the formation of HF upon degradation have been observed for other fluorinated alkylphosphates. Most notably, some stockpiles of sarin in the US arsenal require stabilizers such as tributylamine or diisopropylcarbodiimide to mitigate the corrosion of metal storage containers and munitions by scavenging the acid generated when the parent compound hydrolyzes [14]. Similarly, these types of compounds might act as stabilizers of DFP when stored in glass containers by neutralizing the HF generated as the material degrades, thus preventing autocatalytic degradation (Figure 9) from occurring.

## 4. Conclusions

Proper storage of DFP for use in toxicological evaluations is critical. Degradation of DFP is likely to elicit a concomitant reduction in overall toxicity since the primary degradation product identified in this study, DIPP, has previously been shown not to inhibit cholinesterase activity [15]. It is therefore important to ensure appropriate storage of DFP in order to retain potency so that toxicity values are accurate and there is consistency among toxicology studies across laboratories.

For this study, high purity DFP was synthesized using a one-pot approach by fluorinating the associated phosphite with a mixture of KF and 1,3-dichloro-5,5-dimethylhydantoin. The synthesized material showed minimal (≈4%) degradation when stored at 4°C and no degradation when stored at −10°C or below through 393 days. Conversely, significant degradation was observed in commercially acquired DFP when stored at the manufacturer-recommended storage temperature of 4°C and minor degradation (≈5%) when stored at −10°C within the same time period.

Based on these results, DFP should be stored at −10°C or below to ensure long-term chemical stability. Storage above this temperature would likely result in premature degradation and surreptitiously impact results generated from the use of the material. Additionally, alternatives to glass storage containers and incorporation of stabilizers should be considered.
